# Understanding the relationship between pet owners and their companion animals as a key context for antimicrobial resistance-related behaviours: an interpretative phenomenological analysis

**DOI:** 10.1080/21642850.2019.1577738

**Published:** 2019-02-18

**Authors:** A. Dickson, M. Smith, F. Smith, J. Park, C. King, K. Currie, D. Langdridge, M. Davis, P. Flowers

**Affiliations:** a Safeguarding Health through Infection Prevention [SHIP] Research Group, Glasgow Caledonian University, Glasgow, Scotland; b Faculty of Arts and Social Sciences, Open University, Milton Keynes, England; c School of Social Sciences, Monash University, Melbourne, Australia

**Keywords:** Anti-microbial resistance, affectionate relationships, interspecies behaviours, behaviour change intervention, interpretative phenomenological analysis

## Abstract

**Objectives:** Drivers of antimicrobial resistance (AMR) are diffuse and complex including a range of interspecies behaviours between pet owners and their animals. We employed interpretative phenomenological analysis (IPA) to explore the relationship between pet owners and their companion animals in relation to AMR. **Design:** Cross sectional, qualitative study. **Methods:** Semi-structured interviews were conducted with twenty-three British pet owners, transcribed verbatim and subjected to Interpretative Phenomenological Analysis (IPA). **Results:** Three, inter-related Superordinate themes are presented 1) ‘They’re my fur babies’: unconditional love and anthropomorphism; 2) ‘They share everything with you’: affection and transmission behaviours; and 3) ‘We would err on the side of caution’: decision making and antibiotic use’. **Conclusions:** Affectionate behaviours between companion animals and their owners pose a risk for AMR transmission but they are so deeply treasured that they are unlikely to be amenable to change. In contrast, the promotion of appropriate antibiotic stewardship for pet owners and vets may offer a viable pathway for intervention development, benefitting from synergies with other interventions that target prescribers.

## Introduction

Revolutionary improvements in medical practice in the twentieth century were largely facilitated by antibiotics. Antibiotics are used for both the prevention (prophylaxis) and treatment of infectious diseases. Now however, an increased variety of microbes are displaying resistance to many classes of antimicrobial drugs (European Centre for Disease Prevention and Control, 2016; Holmes et al., 2016; Roca et al., 2015). Antimicrobial resistance (AMR) has been identified as a major world health crisis (Jasovsky, Littmann, Zorzet, & Cars, 2016; World Health Organisation, 2007) threatening to negate decades of medical progress. AMR will lead to substantial increases in untreatable bacterial infections which were previously routinely and effectively treated with antibiotics (Laxminarayan, Sridhar, Blaser, Wang, & Woolhouse, 2016). As a result, resistant organisms reduce treatment efficacy, prolong hospitalisation, increase treatment and post-disease care costs (Umber & Bender, 2009) and increase morbidity and mortality of affected individuals. AMR, therefore, poses a severe threat to human health (Travis et al., 2014).

Intervening effectively with the drivers for AMR is challenging due to their diffuse, inter-professional, interdisciplinary and interspecies nature (Flowers, 2018; Holmes et al., 2016). Such drivers include an increased consumption of antibiotics (Aryee & Price, 2015; Holmes et al., 2016; Martínez-González et al., 2017; Rawson et al., 2016; Rawson et al., 2017; van de Sande-Bruinsma et al., 2008; Wong et al., 2015), inappropriate prescribing behaviour by healthcare professionals (Aryee & Price, 2015; Martínez-González et al., 2017; Rawson et al., 2016; Wong et al., 2015), poor antibiotic stewardship amongst health professionals (Martínez-González et al., 2017; Rawson et al., 2017) and poor compliance with antibiotic treatment amongst the public (Allison, Higginson, & Martin, 2017; Gualano, Gili, Scaioli, Bert, & Siliquini, 2015). The potential zoonotic transfer of microbes between food animals and humans is also a growing area of concern (Broadfoot, Brown, Healey, & Vidal, 2017; Phillips et al., 2004; Wegener, Aarestrup, Gerner-Smidt, & Bager, 1999; World Health Organisation [WHO], 2014). However, to date, the relationship between humans and their companion animals as facilitators of both AMR and of AMR transmission, has largely been overlooked (Barber, Miller, & McNamara, 2003; European Commission, 2017; Holmstrup & Klausen, 2016; Hughes et al., 2012; Pomba et al., 2017; Prescott & Boerlin, 2016; Scott Weese, 2008; Van Balen, Landers, Nutt, Dent, & Hoet, 2017; World Organisation for Animal Health, 2016). To date there has been a lack of epidemiological surveillance to monitor and understand the relative importance of these transmission routes although such evidence is now beginning to emerge, acknowledging companion animals as a source of multi-drug resistant pathogens for humans and proposing a One Health approach to address AMR in both humans and animals (European Commission, 2017; Prescott & Boerlin, 2016; World Organisation for Animal Health, 2016).

One critical aspect of the drivers of AMR between humans and their companion animals is their close physical contact and the shared environment within the household (Committee for Medicinal Products for Veterinary Use, 2015). Close relationships with companion animals play a key role in improving human health both physically and psychologically (Barker & Wolen, 2008; Friedmann & Son, 2009). Potential explanations for such benefits include companion animals as a constant source of attachment security (Beck & Madresh, 2015); companion animals as ‘social catalysts’, enhancing social interactions with other people and thus promoting an indirect effect on wellbeing, alleviating feelings of loneliness and isolation (McNicholas & Collins, 2000); companion animals as reducing perceptions of stressful events (McNicholas et al., 2005); and pets offering companionship which mirrors that of human relationships, fostering positive mental health on a daily basis (Wilson et al.^1998^). Indeed, a review by McNicholas et al. (2005) highlights that over 90% of pet owners consider their companion animal as a valued family member and report that the emotional bond between the pet owner and their companion animal can be as intense as that experienced in many human relationships, facilitating similar psychological benefits.

The powerful bond between pet owners and their companion animals may, therefore, serve as a driver for reciprocal affectionate behaviours between humans and their companion animals. While these affectionate behaviours physically and psychologically benefit pet owners, they also have the potential to contribute to increased interspecies transmission of microorganisms including those which are drug resistant (Wieler, Ewers, Guenther, Walther, & Lubke-Becker, 2011). Such transmission occurs particularly with microorganisms residing on the skin, and mucosal surfaces (Walther et al., 2012) or in faecal matter (Ghasemzadeh & Namazi, 2015; Guardabassi, Schwartz, & Lloyd, 2004). These organisms (e.g. *Staphylococcus aureus*) can be drug resistant or carry resistance mechanisms. Close contact and affectionate behaviours between pet owners and their companion animals, including kissing, petting and stroking are, therefore, a potential risk factor for interspecies transmission of microorganisms and serve as a catalyst for AMR transmission (Lloyd, 2007; Walther et al., 2012; Westgarth et al., 2008).

In addition to the importance of a acquiring a better understanding the transmission behaviours of AMR, it is also imperative to engage with the drivers of resistance within companion animals in the first place. Prior to pet owner-companion animal transmission, there are other important AMR-drivers that relate to (the broad use of) antibiotics. Pet owners have substantial influence over veterinary decision making on antibiotic use, and have been reported to (at times) overrule biomedically informed vet decisions concerning prescribing (Adams & Frankel, 2007; Mateus, Brodbelt, & Stärk, 2011; Smith et al., 2018). Prescribing of antibiotics therefore proves to be a complex series of interactions between the pet owner and vet which are heavily influenced by owner relationships with their companion animals (Mateus, Brodbelt, Barber, & Stark, 2014; Smith et al., 2018).

The present study sought to explore pet owners perspectives on their relationship with their pets and various interconnecting concomitant behavioural domains which drive AMR and AMR transmission. It examines the meaning of affectionate behaviours with companion animals and subsequently explores the emotional and relational catalysts of such behaviours as antecedents for AMR transmission behaviours and antibiotic decision making.

To our knowledge, IPA has not yet been utilised as a method of enquiry for exploring AMR transmission-related behaviours. This is surprising given IPA’s intention to explore *how* a particular participant experiences a particular phenomenon, offering a rich, detailed insider’s perspective of the phenomenon under investigation. The inductive data collection process coupled with a rigorous analysis derives themes from the data itself, as opposed to categorising data on the basis of pre-determined categories or a priori assumptions. While IPA is both applicable and useful in a variety of research topics, it lends itself to research within the field of Health Psychology (Brocki & Wearden, 2006). Given the novel, experiential, inductive and idiographic focus of the current research, IPA was considered as an ideal methodological fit. In this study, IPA ‘gives voice’ to pet owners and affords a richer, deeper understanding of their relationships with companion animals and they ways in which these relationships potentially fuel AMR related risk behaviours.

## Materials and methods

### Participants

Participants were twenty-three British pet owners (16 females and 7 males). The age of participants ranged from 32 to 77 years. Participants were resident in various locations in Great Britain; 17 participants resided in Scotland and 4 in England. With regards to companion animal ownership, 13 were dog owners, 4 were cat owners and 3 were rabbit owners. A further 3 participants owned a combination of dogs, cats or rabbits. For the purpose of this paper, companion animals are defined as small domestic animals that cohabit and share close physical contact with humans (King et al., 2017). The three most popular species in the UK were chosen as inclusion criteria (e.g. dogs, cats and rabbits) (Pet Food Manufacturer’s Association, 2017). Table 1 below, provides further participant details.

**Table 1.** Participant details.

Pseudonym	Age	Gender	Geographical location	Method of interview	Type of animal	Number of animals
Lady	25–59	Female	Scottish Central Belt	Phone	Dog	2
Monty	25–59	Female	Scottish Central Belt	Phone	Dog	1
Laura	25–59	Female	Scottish Central Belt	Phone	Cat	2
Tom	25–59	Male	Scottish Central Belt	Phone	Dog	1
Kereen	25–59	Female	Scottish Central Belt	F2F	Dog	1
Bob	25–59	Male	England	Phone	Dog	1
Pete	25–59	Male	Scottish Central Belt	F2F	Dog	1
Stephen	25–59	Male	Scottish Central Belt	F2F	Dog	1
Maura	60+	Female	England	Phone	Mix	2 dogs, 1 cat
Sarah	25–59	Female	England	Phone	Mix	1 dog, 1 cat
Splash	25–59	Female	England	Phone	Dog	1
Rosemary	25–59	Female	Scottish Central Belt	F2F	Dog	2
Frank	25–59	Male	Scottish Central Belt	F2F	Dog	1
Dave	25–59	Male	Scottish Central Belt	F2F	Cat	2
Karen	25–59	Female	Scottish Central Belt	F2F	Cat	1
Jason	25–59	Male	Scottish Central Belt	Phone	Dog	2
Matilda	25–59	Female	Scottish Central Belt	F2F	Rabbit	2
Valerie	25–59	Female	Scottish Central Belt	Phone	Dog	1
Kerry	60+	Female	Scottish Central Belt	Phone	Dog	2
Isabella	25–59	Female	Scottish Central Belt	Phone	Rabbit	2
Georgina	25–59	Female	Scottish Central Belt	Phone	Cat	1
Penelope	25–59	Female	Scottish Central Belt	Phone	Mix	2 rabbits, 1 cat
Barbara	25–59	Female	Scottish Central Belt	Phone	Rabbit	5

Note: FTF: Face-to-face.

### Procedure and interview

Recruitment occurred via: 1) personal contacts of the research team; 2) social networking among recruited participants; 3) adverts on social media (Facebook and Twitter); and convenience sampling at a local veterinarian practice. Volunteer participants were required to meet the following inclusion criteria: 1) ownership of at least one companion animal, either a dog, cat or rabbit; 2) registration of the animal at a recognised veterinary practice; 3) receipt of prescribed antibiotics for their companion animal, on at least one occasion from their registered veterinarian practice; and 4) no recent companion animal bereavement. All participants received an information sheet which detailed the nature of the study and what would be required. The contact details of the lead researcher were detailed herein to enable interested individuals to express their willingness to take part. Semi-structured interviews were then arranged at a time and location which was most convenient for the participant. All participants were provided the option of conducting the interview face to face (at a location of their choice) or via telephone. Eight interviews were conducted face to face and 15 were conducted via telephone. Interview duration varied between 28 and 68 min and all interviews were audio recorded and transcribed verbatim. All participants provided written consent prior to the interview commencing.

In line with recommendations by Smith, Flowers, and Larkin (2009) a broad topic guide was developed and operationalised flexibly with open ended questions designed to be non-directive and participant-led. Typical questions included ‘Tell me about your relationship with your companion animal’, ‘What does your companion animal mean to you?’, ‘Tell me about a time when your companion animal was unwell and required antibiotics’ and ‘How do you feel about giving your companion animal antibiotics?’ The interview broadly aimed to explore the participant’s experiences and behaviours in relation to their pet, healthcare and antimicrobial resistance. Interviews were inductive and encouraged the participant to identify key experiences they believed to be important. There was no financial recompense for participation in the study.

## Analysis

### Interpretative phenomenological analysis

Interviews were subjected to IPA. The process of analysis involved several key stages, as detailed in Smith et al. (2009). Each transcript was read repeatedly in order to increase familiarity. Key words, phrases or idiosyncratic figures of speech were highlighted and emerging themes were documented and clustered into groups. The process was repeated for the remainder of the transcripts. All transcripts were then further analysed in order to highlight similarities and differences within the group. The data was then clustered into thematic categories in order to identify superordinate and related sub-themes. The extracts presented herein have been selected as they best encapsulate the essence of the theme. Pseudonyms are used throughout to protect the anonymity of the participants.

### Ethics Statement

Ethical approval was granted from the University ethics committee for the Department of Health and Life Sciences at Glasgow Caledonian University (Reference number: HLS/NCH/16/001).

## Results

Three inter-related superordinate themes are presented. Each addresses context-bound antimicrobial resistance-related behaviours and concomitant antecedents: 1) ‘They’re my fur babies’: ‘unconditional love and anthropomorphism’; 2) ‘They share everything with you’: affection and everyday transmission behaviours; and 3) ‘We would err on the side of caution’: decision making and antibiotic use’. Thus the themes which follow trace the arc of the relationship between pet owners and their companion animals to illuminate *how* such relationships have the potential to lead to both AMR-transmission behaviours and antibiotic decision-making behaviours.

### ‘They’re my fur babies’: unconditional love and anthropomorphism shape interspecies interactions

The first superordinate theme explores the deeply emotive nature of the pet owner-companion animal relationship. Companion animals were reported to provide company, security, loyalty and companionship for pet owners. This ‘unique’ and ‘special’ relationship was spoken of in terms of a sense of ‘*incredible affection’*, unconditional love and anthropomorphism. Many of the participants described their companion animal relationship as parental, likening their animals to children. Many participants described themselves as ‘*mummy*’ to their pets, described their pets as their ‘*babies*’, ‘*toddlers*’ or ‘*children*’ and considered their companion animal as ‘*family*’ or ‘*best friends*’. At the very heart of these emotive relationships with companion animals was a belief in reciprocal, unconditional love which many participants highlighted as being entirely unique to the bond they shared with their companion animal:

It’s the unconditional love that you get from them [dog] and they’re always happy to see you. You know, you can have the worst day ever and you could go out the house for kinda you know, leave the room and go to the loo and when you come back they’re wagging their tail because they’ve missed you. You know, who else do you get that from? (Kereen)

Kereen’s extract encapsulates the unqualified, absolute love she shared with her dog- this is a love which appears to have no bounds and no end. Reflecting many of the participants’ accounts, her extract identifies their relationship as being one of transcendent love that could not be matched in relationships between humans. Unreserved, unadulterated affection from companion animals appeared to unleash parental-like emotions and the assumption of carer responsibility in many of the participants. This parental, animal care occurred both in participants’ who were childless at the time of interview as well as in those who were parents:

She [dog] does the whole unconditional love thing … . (…) and that unlocks some kind of- I don’t have kids- so unlocks some kind of paternal feelings in me that I had no idea were even there. (Frank)

and

He’s [dog] part of the family. We consider him as our firstborn child. (Tom)

It appeared that for many participants, intensely affectionate relationships with companion animals offered a powerful experience of love where their companion animals were considered to be entitled to family membership. These emotions were undoubtedly marked by, and deepened through, pronounced anthropomorphism:

I’m one of these nutty people that don’t see pets as animals, I see them as extensions of the family and as people and they’re my babies. (… .) They’re like little people to me (…)They are a member of my family and as I would defend, and protect and care and love any of my human family, it is as, I feel the same about my pets. And actually, perhaps more so because I’ve chosen to have that relationship. (Maura)

Maura expressed an awareness that such personification of companion animals is foolish, yet like her, almost all participants in the current study subscribed to anthropomorphism of their beloved pets. Companion animals were considered to ‘*complete a family’*, and to be ‘*central’* or ‘*core*’ to the family unit. Undeniably, the affective dimensions of the meaningful, unconditional relationships participants shared with their companion animals served as primary antecedents to tactile behaviours which present interspecies transmission routes for AMR, as discussed in what follows.

### ‘They share everything with you’: affection and everyday transmission behaviours

The unconditional nature of the companion animal-pet owner relationship and its associated anthropomorphism led to a series of affectionate behaviours between the pet owner and their companion animals. These included licking (described as ‘*doggy kisses’*), stroking, grooming, cleaning, sleeping, feeding and hugging. The meaningfulness of these affectionate behaviours is best captured in the following extract by Kereen:

He’ll [dog] come and … instead of sitting over on his own, or on the floor or whatever, he’ll come over and kind of snuggle in beside me on my seat. Sometimes he’ll just come and sit and lick my hand for ages and kind of like he does this funny wee thing, and he only tends to do it with people that he’s very close to. Hell kind of lick your hand and then just hold it between his teeth, so he doesn’t bite but he just holds your hand between his teeth and then just gives a few wee licks. Things like that, you know, just seem to be his way of showing his affection. (Kereen)

There is a strong sense of every day intimacy within participant accounts of these behaviours. They are understood to represent closeness, connection, acceptance and belonging. The notion of ‘hand holding’ signifies the sharing of personal boundaries and perhaps represents a shared sense of comfort, protection and safety. Peppered across the participants’ accounts is a sense of these physically intimate relationships promoting a feeling of ‘togetherness’. For many participants, togetherness extended to sharing personal space, including bedrooms or the bed itself:

One of them (rabbits) behaves like a dog. So he’ll come up and sleep under the bed which is bizarre. And he’ll jump up on your lap when he’s in the mood to snuggle and you can pat him and everything. … But I think it’s the interaction isn’t it? (Matilda)

and

Sometimes in the cold weather I’ll wake up in the night and she’s (cat) just like, crawled up and burrowed under the duvet, and she’s just decided ‘right, if it’s good enough for him, it’s good enough for me. (Dave)

Again, both of these extracts convey a strong sense of connectedness, exchange and comfort. The pet owner-companion animal relationship is made meaningful and enacted through close physical contact, affection and displays of emotion. It is as though the owner and companion animal are side-by-side in life, bound by their unique and unconditional bond.

Other physical displays of affection included ‘cuddles’, and being woken in the mornings by ‘kisses’. Many participants also reported sharing eating utensils (e.g. cups and plates) or allowing their companion animals to eat from their bare hands:

I’m one of these awful people that I will let them lick my plate and things like that … . you’re not meant to let them lick your plate, are you? (Maura)

and

They [rabbits] used to follow me to a cupboard where their food was kept, get some food out my hand, they'd eat off my hand. If I lie down on the floor they jump on my back, so incredibly endearing and they’re just so cute and they are the softest animals I’ve ever stroked. (Isabella)

Maura’s account perhaps reflects an acknowledgment of the potential zoonotic risks associated with her ‘sharing’ behaviour with her companion animals. However, it might also reflect social norms relating to perceived hygiene. For her and the other participants, this is related to the transmission of microbes in general and not to antimicrobial resistance in particular. The expression of the bond that pet owners share with their animals diminishes any motivation for behaviour change. Maura identifies herself as being ‘one of these awful people’, signifying awareness that her hygiene practices are not normative- perhaps identifying membership of a distinct category of animal lovers who endorse and share similar transgressive behaviours and relationships with their pets. Similarly, Isabella identifies a gravitational pull towards her companion animals- in fact, she herself enters their personal space (the floor), to display and reciprocate affection. While such reciprocal affection appeared to be cherished by all participants in the current study, they are also potential transmission behaviours for microbes and potential enablers for the interspecies transmission of drug resistance microbes. Moreover, anthropomorphism and emotional attachment were antecedents of antibiotic decision making which we highlight in the following section.

### ‘We would err on the side of caution’: decision making and antibiotic use

Amongst the participants, knowledge of AMR was poor and understanding of interspecies transmission of AMR organisms was largely absent. Participants did demonstrate an awareness of problematic antibiotic use. At times this was in relation to AMR risk, but more commonly it was in relation to the notion that antibiotics could damage organs (i.e. the liver) or that the recipient of antibiotics could develop resistance to them:

All the bits and bobs I’ve heard from mainstream media of, you know, people and animals building up a resistance and the viruses building up a resistance to the antibiotics. (Rosemary)

It is against this background that the participants spoke of decisions relating to the management of their pets health. Just as ‘unique’, ‘parental’, ‘unconditional’, affectionate relationships shaped transmission behaviours, feelings of responsibility and ‘protectiveness’ of their companion animals, shaped pet owner behaviour when their animals required veterinarian attention. Participants highlighted that in such situations, *they*, with their intimate knowledge of their animals, ultimately carried the burden of ‘detecting’ or ‘reading the signs’ and appropriately gauging whether veterinarian attention and/or antibiotics were required. Pet owners often reported species-specific attitudes towards antibiotic use. Many reported refuting antibiotics personally but actively seeking, or welcoming their use for their companion animals:

I’ve got some sort of, eh, insect bite and its definitely infected and then you know, my Mum’s going ‘oh you need antibiotics for that’ and I’m going ‘oh no its fine’ (laughter) you know? Em, but then obviously when it comes to eh, you know, your pets it seems to be completely different for some reason (laughter). I don’t know why, that’s what I say, I freely admit its some kinda, I’m kinda been a bit, I’m kind a like ‘no, I won’t take antibiotics’ but then obviously when it comes to your pet you’re going ‘oh no, definitely’. (Laura)

Laura’s pithy quote suggests an almost involuntary, instinctive and automatic decision in welcoming antibiotics for her companion animal. This instinctive response, however, is juxtaposed with a prolonged process of deliberation and delay in decision making around personal antibiotic use. For many participants this clarity and expediency in decision making for animal care was predicted by a fear that delay or complacency would have serious consequences for their companion animal. As such, most, in direct contrast to decisions to use antibiotics on themselves, chose to ‘err on the side of caution’ and administer antibiotics, often as a ‘protective’, better-safe-than-sorry approach to the risk of infection:

I think they’re almost like children in a way so if they start to be ill you’ve got to react quite quickly. Because they can go down quite quickly and because I can’t tell, they can’t speak to me. (Matilda)

Perceived vulnerability of the companion animal appears to underpin Matilda’s decision making. An overwhelming sense of moral responsibility is evident here- the companion animal depends on Matilda since it cannot make its own choices and therefore exercise its own agency and consent over health decisions. Matilda, like many other participants in our research, perceived herself as being required to make the ‘right’ decision on behalf of their animals. The gravity of the decision was emphasised in many of the participant accounts as they themselves were not prepared to live with the consequences of making the ‘wrong’ decision (e.g. refusing antibiotics and living with the potentially fatal consequences). As many participants highlighted, they simply ‘*couldn’t imagine living life without their pet*’ (Monty).

As Matilda’s excerpt (above) highlights, the companion animal’s inability to communicate foregrounded the heightened sense of responsibility owners felt towards their pets and therefore, their decision to seek and/or accept antibiotics as a preventative measure:

You’ve got the responsibility that you get, I mean they can’t, you know, stating the obvious, but they can’t really communicate to you what’s going on, so I think it’s just maybe, em, you kind of err on the edge of caution, I suppose. Maybe if it was someone who could communicate to you, but they can’t, so with that kinda responsibility I think, no, I’ll just, you know, I’ll just maybe do more than I maybe would, you know? (Laura)

It seems that the pet owners considered themselves as the guardian of their pets, speaking for them in decisions around health, risk and treatment. It was therefore *their* responsibility to protect, guard and care for their animals by adopting precautionary antibiotic action. An alternative approach of observation and monitoring of the companion animal’s condition, a ‘wait a see’ approach, was deemed to be just ‘too risky’:

Its obviously just the kinda guilt thing that’s obviously in your head you’re thinking ‘oh right, my cat’s got an infection, we’ll see how it goes’ and then you know, they might die, that’s kind of worst case scenario and they might think ‘oh, if only I’d got antibiotics it would be ok. (Laura)

## Discussion

This paper is the first of its kind to explore the meaning of affectionate human-companion animal relationships and its importance in understanding the determinants of a range of AMR-related behaviour. While our findings further support those of previous research highlighting the intense emotional bond (McNicholas et al., 2005) and companionship (Wilson, Turner, Collis, & McNicholas, 1998) between pet owners and their companion animals, it is novel in identifying the importance of this relationship in shaping subsequent behaviours that are central to reducing the drivers of AMR. Thus through an in-depth inductive analysis of participants’ accounts, we have gained a deeper understanding of the overall context and associated behavioural system connecting people to their pets. We have shown the ways that multiple AMR- related behavioural domains are understood within interspecies and emotive, relational contexts. We propose that such AMR-related behaviours should be the foci for future intervention design to reduce AMR amongst the pet owning public.

Previous research has shown affectionate behaviours between pet owners and their companion animals can lead to AMR transmission (Guardabassi & Prescott, 2015; Lloyd, 2007) but they have predominantly focussed on the nature and level of antibiotic resistance rather than on an understanding of what drives such behaviours. Our analysis extends this knowledge by providing a phenomenologically oriented account illuminating the relational context and the ways in which it drives a range of behaviours associated with AMR.

Participants understood that engagement in some behaviours such as sharing saliva and skin-to-skin contact was unhygienic and socially sanctioned yet they did not understand these behaviours as potential drivers of AMR. Some participants were aware of issues surrounding inappropriate antibiotic use, although overall across the participants their understanding of problematic antibiotic use was incomplete and varied. Further, our participants understood AMR-related behaviours to be deeply embedded within routine family life in the domestic sphere. Such behaviours are experienced by pet owners as ordinary, everyday expressions of closeness and love. This novel finding is important as it not only highlights emotion as a powerful driver for zoonotic AMR transmission behaviours but also illuminates *why* people engage in such behaviours and *why* such behaviours may not be amenable to change.

From a more behavioural perspective, our analysis also highlights that diverse behaviours (stroking, kissing, taking animals to the vets, asking for antibiotics, compliance with antibiotics) shared key antecedents. First and perhaps foremost, poor knowledge and understanding of the mechanisms of AMR and potential transmission routes reduced psychological capability to engage with any ideas relating to behaviour change. Secondly, powerful feelings of unconditional love, duty of care and responsibility characterised the ways in which participants thought of their companion animals and shaped AMR-related behaviour. Meta-theoretical frameworks such as the Theoretical Domains Framework (TDF) (Cane, O’Connor, & Michie, 2012) would understand these behavioural determinants as relating primarily to emotions and reinforcement, or within the COM-B model of behaviour change (Michie et al., 2013) as being primarily motivational (both automatic and reflective).

Our participants’ spoke of how these powerful feelings shaped their AMR related decision-making; participants would avoid antibiotics for themselves but would gladly accept them for their pets. Pet owners tended to project worst-case scenarios, anticipating feelings of intolerable guilt in living with the devastating consequences of potentially losing a pet through their complacency. The inability of their companion animals to communicate the specificity and severity of their symptomatology prompted pet owners to take precautionary and preventative measures to protect their pets. Such measures were acknowledged (at times) to be unwarranted. A ‘better safe than sorry’ rationale for potentially unnecessary antibiotic use may have reduced pet owners’ immediate anxieties, but at the same time, serves as a driver to facilitate AMR. These findings highlight the importance of future intervention content to directly address these antecedents. They also support previous calls for interventions to address the pet owning public’s knowledge and understanding of AMR (European Commission, 2017; Prescott & Boerlin, 2016; World Health Organisation for Animal Health, 2016). Equally, our analysis suggests that behaviour change techniques and the wider intervention content should address the powerful motivational, and specifically the emotional, determinants of AMR-related behaviours.

Finally, in relation to thinking about intervention development, we consider our results as potential foci for future behaviour change interventions. Our analysis has shown a variety of AMR-related behaviours within a particular interspecies context. A cluster of highly related behaviours form a non-verbal means of communicating and maintaining relationships between people and their pets. These include everyday affectionate behaviours such as stroking, grooming and petting (Prescott & Boerlin, 2016; Walther et al., 2012; Westgarth et al., 2008) commonly reported behaviours such as kissing and licking (Prescott & Boerlin, 2016); and less frequently reported behaviours such as sharing food and drink utensils. A distinct set of behaviours were also reported in relation to interactions with veterinarians and the use of antibiotics. In considering potential targets of future behaviour change interventions we believe the latter behaviours form a more suitable basis for intervention development for a number of reasons. First, there exists a growing literature regarding pet therapy and the positive health benefits associated with many of the intimate behaviours outlined by our participants (Amiot & Bastian, 2015; Barker & Wolen, 2008; Friedmann & Son, 2009; Haraway, 2003). Second, given the centrality of these behaviours as the very expression of the nature of the relationship between pets and pet owners, our findings suggest it is highly unlikely that these behaviours are easily amenable to change. The overall psychosocial cost of changing these behaviours would be too high. Third, given the current low population prevalence of pets and people colonised with AMR, the net benefit of changing these behaviours may be low. As highlighted elsewhere (De Briyne, Atkinson, Pokludova, Borriella, & Price, 2013; Guardabassi & Prescott, 2015; Weese et al., 2015), rapid diagnostic tests are key and we propose here that they may also enable the specific targeting of family units in which one member is colonised and local behaviour change interventions could be implemented therein.

In contrast to these major barriers to interventions focusing on transmission behaviours, interventions that focus upon appropriate antibiotic use seem far more promising (Aryee & Price, 2015; Huttner, Harbrath, & Nathwani, 2014; Robilotti et al., 2017). Key antibiotics (including Penicillins and Tetracyclines) are shared between humans and the animals with which they have close relationships with (HM Government, 2015). While a One Health strategy to increase the surveillance and minimise the inappropriate use of antimicrobials (particularly those considered to be critically important) in humans and animals has been implemented (WHO, 2017), we suggest that such interventions could dovetail with wider psychosocial interventions addressing the behaviour of the public and antibiotic prescribers within human health care. As our analysis has highlighted, there are conundrums with the public’s understanding and engagement with AMR and how it relates to humans, let alone how it may relate to pet health or indeed One Health (Mattar et al., 2016; Reeve-Johnson, 2017; Robinson et al., 2016). The promotion of more appropriate antibiotic stewardship in both vets (who prescribe the antibiotics) and pet owners (who administer them) may pave the way forward.

However, there a number of potential challenges to overcome. Currently, there is little in the way of understanding, or guidance for appreciating the developing risks of AMR pathogens in companion animals (Day, 2016). For example, the European Medicine’s Agency (EMA) presently has no guidelines for the risk of transmission of AMR due to contact between companion pet and pet owners (Pomba et al., 2017). Guidelines for ensuring the appropriate use of antibiotics are available, but emphasise the food animal populations. More appropriate antibiotic stewardship guidelines need to be implemented for pet animal populations specifically (Mateus et al., 2011). The British Veterinary Association (British Veterinary Association, 2017) and British Small Animal Veterinary Association (British Small Animal Veterinary Association, 2017) further emphasise the need for appropriate stewardship by vets when stating that vets should only use narrow spectrum antibiotics that are effective against specific organisms.

Within this paper, we have highlighted the emotional antecedents of AMR transmission behaviours, *what* prompts these behaviours to occur and detailed *how* behaviours are embedded within wider relationship contexts. In doing so, we have identified the active components of species-specific decision making and how this drives often inappropriate antibiotic use in pet owners. Herein, we have developed a rich understanding of the key behaviours which potentially serve as drivers for AMR in the pet owning public, as identified by pet owners themselves.

We suggest that IPA as a method of enquiry may be a useful preliminary tool to inform a secondary, behavioural and theoetical exploration of novel, complex behavioural domains. It also works well at inductively generating the identification and understanding of possible mechanisms of behaviour change. This paper has demonstrated that the use of IPA enables a richly nuanced, in-depth, inductive exploration of behaviours within contexts, in this case pet owners and their lived experiences with companion animals. These approaches to intervention development are particularly important when there is no available evidence base to assist with intervention development.

It is important to acknowledge that there may be a sample bias in the present study in that only those participants who chose to volunteer took part. It is possible that those pet owners who share a particularly special bond with their pets would have volunteered to partake as opposed to other pet owners who do not share this relationship. The sample also represented a predominantly middle-class, well-educated demographic. Finally, we have only recruited owners of dogs, cats and rabbits (whose pets reside within the home) in this study (the three most popular domestic pets). Our findings, therefore, reflect the experiences of these particular participants; although they may (with caution) be generalised to the wider pet owner/vet population. Further research with random samples may be warranted to secure wider population representativeness. Further research could also explore the behavioural drivers, decision making processes and inter-personal relationships between veterinarians and pet owners and their influence on antibiotic use in companion animals. Despite these limitations, this study offers: 1) valuable insight into the potential relational catalysts for AMR; 2) identifies key antecedents amenable to change in future behaviour change intervention; and 3) highlights IPA as an innovative, insightful method of inquiry particularly useful for early stages of research informing behavior change intervention design and development.
